# Efficacy and Safety of Ceftazidime-Avibactam for the Treatment of Carbapenem-Resistant *Enterobacterales* Bloodstream Infection: a Systematic Review and Meta-Analysis

**DOI:** 10.1128/spectrum.02603-21

**Published:** 2022-04-04

**Authors:** Yan Chen, Hui-Bin Huang, Jin-Min Peng, Li Weng, Bin Du

**Affiliations:** a Medical Intensive Care Unit, State Key Laboratory of Complex Severe and Rare Diseases, Peking Union Medical College Hospital, Peking Union Medical College and Chinese Academy of Medical Sciences, Beijing, China; b Department of Critical Care Medicine, Beijing Tsinghua Changgung Hospital, School of Clinical Medicine, Tsinghua University, Beijing, China; University of Texas Southwestern Medical Center

**Keywords:** ceftazidime-avibactam, carbapenem-resistant *Enterobacterales*, bloodstream infection

## Abstract

Several clinicians use ceftazidime-avibactam (CAZ-AVI) to treat bloodstream infections (BSIs) due to carbapenem-resistant *Enterobacterales* (CRE), although no conclusive data support this practice. We aimed to assess the efficacy and safety of CAZ-AVI in the treatment of CRE bacteremia. PubMed, Embase, and Cochrane Library were systematically searched until 5 November 2021. Studies comparing the clinical outcome of CAZ-AVI with other regimens in CRE BSI were included if they reported data on mortality. Results were expressed as risk ratios (RRs) or mean differences with accompanying 95% confidence intervals (95% CIs). Eleven articles with 1,205 patients were included. CAZ-AVI groups showed a significantly lower 30-day mortality than control groups of other regimens (RR = 0.55, 95% CI of 0.45 to 0.68, *P* < 0.00001). The result is robust when a colistin-based regimen serves as the control group (RR = 0.48, 95% CI 0.33 of 0.69, *P* < 0.0001). In subgroup meta-analyses, the 30-day mortality was significantly lower in patients infected with CRE producing Klebsiella pneumoniae carbapenemase (RR = 0.59, 95% CI of 0.46 to 0.75, *P* < 0.0001). Additionally, patients in CAZ-AVI groups had a significantly higher clinical cure rate (RR = 1.75, 95% CI of 1.57 to 2.18, *P* < 0.00001) and lower nephrotoxicity rate (RR = 0.41, 95% CI of 0.20 to 0.84, *P* = 0.02). No significant differences of relapse rates were demonstrated in 2 groups (RR = 0.69, 95% CI of 0.29 to 1.66, *P* = 0.41). Although the current study is based on observational studies with a small sample of participants, the findings suggest that CAZ-AVI treatment is effective and safe compared with other antibiotics, including colistin, in CRE BSI.

**IMPORTANCE** Ceftazidime-avibactam (CAZ-AVI) has been used as a frontline agent in the treatment of multidrug-resistant (MDR) Gram-negative bacterial infections. However, the efficacy and safety of CAZ-AVI on carbapenem-resistant *Enterobacterales* (CRE) bloodstream infections (BSIs) remain unclear. Patients with CRE BSIs were often enrolled in small-sized clinical studies, together with other sites of infections, which reported pooled results. In this meta-analysis, the efficacy and safety were compared between CAZ-AVI and any other regimens used against CRE infections. The findings suggest that patients in the CAZ-AVI group had a significantly lower 30-day mortality than any other regimens and than colistin-based regimens. This paper provides a rationale for the use of CAZ-AVI in one of the most urgent antimicrobial-resistant infections of CRE bloodstream infections.

## INTRODUCTION

*Enterobacterales* are a family of enteric Gram-negative bacilli that include common human pathogens (such as Klebsiella pneumoniae and Escherichia coli) with increasing bacterial resistance (1). Multidrug-resistant (MDR) *Enterobacterales* infections, including carbapenem-resistant *Enterobacterales* (CRE), are associated with significant morbidity and mortality and represent a growing threat to public health worldwide (2). With resistance to carbapenems and most available antibiotics, the optimal clinical management of CRE infections remains to be established (3). As a result, CRE has been listed as one of the three most urgent antimicrobial-resistant threats by the Centers for Disease Control and Prevention (CDC) and as pathogens of critical priority by the World Health Organization (WHO) (1, 4).

Meanwhile, bloodstream infections (BSIs) caused by CRE were associated with worse outcome compared to other sites of infections. According to Xu et al., the pooled mortality was much higher than urinary tract infection (UTI; 54.3% versus 13.52%) in patients with carbapenem-resistant Klebsiella pneumoniae (5). Additionally, approximately 20% of patients with BSIs received inappropriate antibiotic therapy in U.S. hospitals, especially those with infections caused by *Enterobacterales* or Staphylococcus aureus that were resistant to empirical agents (6), leading to increased risk of mortality (7, 8).

Ceftazidime-avibactam (CAZ-AVI) is a novel β-lactam/β-lactamase inhibitor with *in vitro* activity against CRE producing Ambler class A (e.g., Klebsiella pneumoniae carbapenemase [KPC]), class C (e.g., AmpC), and some class D (e.g., OXA-48) β-lactamases (9, 10). CAZ-AVI has been approved by the U.S. Food and Drug Administration (FDA) and the European Medicines Agency (EMA) for infections without additional therapeutic options, such as complicated intraabdominal infections, complicated urinary tract infections, and hospital-acquired pneumonia/ventilator-associated pneumonia (11–13). Therefore, CAZ-AVI has been used as a frontline agent in the treatment of CRE infections.

Recently, CAZ-AVI was recommended as the preferred treatment option for UTIs caused by CRE based on randomized controlled trials (RCTs) by the Infectious Diseases Society of America (IDSA) (14). However, patients with CRE BSIs were often enrolled in small-sized clinical studies, together with other sites of infections, which reported pooled results. Previous meta-analyses regarding the efficacy of CAZ-AVI on CRE infections typically focused less on the particular site infection of BSI due to the lack of available data.

In a meta-analysis of 9 randomized controlled trials and 3 observational studies, CAZ-AVI exhibited a comparable clinical and microbiological response to carbapenems in the management of severe Gram-negative infections (15). Moreover, in the subgroup of CRE infections caused by BSI (140 patients of 2 studies), CAZ-AVI was associated with improved clinical response (risk ratio [RR] = 2.11, 95% confidence interval [95% CI] of 1.54 to 2.88) (15). Recently, several observational studies comparing CAZ-AVI and other antibiotics (comparators) in patients with CRE BSIs have been published. Therefore, we conducted a systematic review and meta-analysis to assess the efficacy and safety of CAZ-AVI for the treatment of CRE BSIs.

## RESULTS

### Study selection.

**FIG 1:**
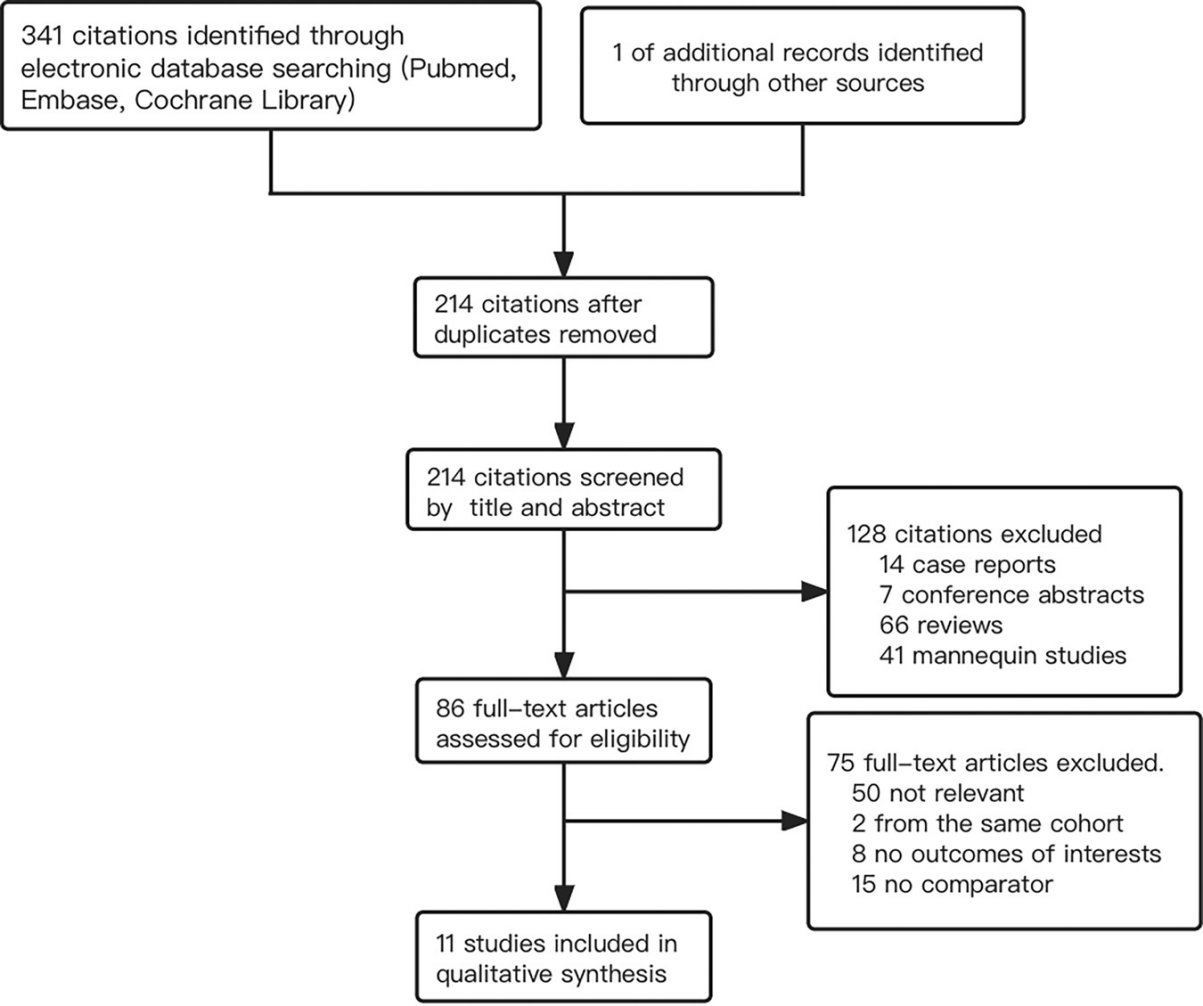
Flow chart of process of literature search and review based on eligible criteria.

Following the outlined search strategy, 341 studies were identified through electronic database searching, and 1 additional record was identified through reference lists. Of these 341 citations, 127 were excluded due to duplicate publication, and 128 were excluded after reviewing the titles and abstracts because of case report (*n* = 14), conference abstract (*n* = 7), review (*n* = 66), and *in vitro* studies (*n* =41). Therefore, 86 full-text studies were assessed for eligibility. Of the 86 studies, 75 were further excluded due to irrelevance (*n* = 50), studies from the same cohort (*n* = 2), no predefined outcomes (*n* = 8), and no comparator (*n* = 15). The remaining 11 studies were included in the final analysis (Fig. 1). The results of quality assessment and funnel plot of publication bias are shown in Tables S1 and S2 and Fig. S1 in the supplemental material.

### Study characteristics.

The characteristics of the included 11 studies are described in Table 1, including 3 prospective and 8 retrospective observational studies (16–26). The outcomes included mortality in 11 studies (1,205 patients), clinical cure in 6 studies (567 patients) (16, 17, 19, 22, 25, 26), relapse in 4 studies (455 patients) (17–19, 26), and nephrotoxicity in 5 studies (380 patients) (16, 17, 19, 22, 26).

**TABLE 1:** Characteristics of included studies

Reference	Study design^*a*^	Region	No. of patients	Population characteristics, *n* (%)^*a*^	Age (yr), mean ± SD	Male, *n* (%)	Site of bacteremia (*n*, %)^*a*^	Pathogens (%)^*a*^	Resistance (*n*, %)^*a*^	Treatment (*n*, %)^*a*^
16	RS	Spain and Israel	31	Hematologic malignancies 31 (100), ICU 3 (10)	59 ± 60	19 (61)	UTI (1, 3), CRBSI (6, 19), primary (14, 45), RTI (6, 19), IAI (2, 6), wound/ulcer (1, 3), other (1, 3)	KPN (81), S. marcescens (6), En. cloacae (3), K. oxytoca (6), Es. coli (3)	KPC (12, 39), OXA-48 (19, 61)	CAZ-AVI (8, 26) versus COL (1, 3) versus others
17	RS	USA	109	ICU 55 (50)	61 ± 16	61 (56)	Primary (28, 26), IAI (50, 46), RTI (14, 13), UTI (13, 12), SSTI (4, 4)	KPN (100)	KPC (106, 97)	CAZ-AVI (13, 12) versus COL (41, 38) versus others Monotherapy (37, 34): CAZ-AVI (8, 7), COL (4, 4)
18	RS	Italy	208	ICU 66 (32)	66 ± 32	144 (69)	NA	KPN (100)	KPC (208, 100)	CAZ-AVI (104, 50) versus COL (19, 9) versus others Monotherapy (49, 24): CAZ-AVI (22, 11), COL (9, 4)
19	RS	Greece	50	ICU 50 (100)	NA	NA	NA	KPN (100)	KPC (72, 94)	CAZ-AVI (22, 44) versus others Monotherapy (8, 16): CAZ-AVI (7, 14)
21	RS	Italy	91	ICU 91 (100)	64 ± 16	65 (64)	CRBSI (13, 13), primary (14, 14), RTI (11, 11), UTI (21, 21), SSTI (18, 18), IAI (24, 24), endocarditis (1, 1)	KPN (100)	KPC (102, 100)	CAZ-AVI (13, 14) versus COL (61, 67) versus others
20	PS	Greece	142	ICU 67 (47)	63 ± 17	95 (67)	UTI (23, 16), non-UTI (119, 83)	KPN (100)	KPC (142, 100)	CAZ-AVI (71, 50) versus others
22	PS	Italy and Greece	102	ICU 35 (34)	70 ± 17	69 (68）	UTI (33, 32), CRBSI (27, 27), SSTI (12, 12), RTI (9, 9), IAI (7, 7)	KPN (81), Es. coli (3), Enterobacter spp. (5), M. morganii (1)	NDM (79, 80), VIM (20, 20)	CAZ-AVI + ATM (52, 51) versus COL (27, 26) versus others Monotherapy (4, 4): CAZ-AVI (0, 0), COL (2, 2)
23	RS	China	89	NA	55 ± 18	65 (73)	Non-UTI or biliary tracts (77, 87)	KPN (100)	NA	CAZ-AVI (9, 10) versus COL (20, 22) versus others
24	PS	China	135	NA	57 ± 18	145 (70)	NA	KPN (86)	KPC (146, 70), NDM (34, 16)	CAZ-AVI (4, 3) versus COL (28, 21) versus others Monotherapy (92, 68): CAZ-AVI (1, 1), COL (3, 2)
26	RS	Saudi Arabia	61	ICU 11 (18)	54 ± 19	36 (59)	UTI (5, 8), RTI (7, 11), IAI (15, 25), SSTI (4, 7), CRBSI (21, 34), infected graft (2, 3)	KPN (79), Es. coli (12)	OX-48 (31, 62), KPC (6, 12), NDM (13, 26)	CAZ-AVI (32, 52) versus COL (29, 48) Monotherapy (9, 15): CAZ-AVI (9, 15)
25	RS	China	187	NA	67 ± 15	115 (62)	CRBSI (53, 28), RTI (45, 24), IAI (43, 23), UTI (34, 18), primary (12, 6)	KPN (88), Es. coli (11)	NA	CAZ-AVI (35, 19) versus COL (103, 55) versus others Monotherapy (32, 17): CAZ-AVI (13, 7)

a NA, not applicable; RS, retrospective study; PS, prospective study; KPN, K. pneumoniae; AG, aminoglycosides, AK, amikacin; ATM, aztreonam; BLIBL, β-lactamase-inhibiting β-lactams; CB, carbapenems; COL, colistin; FOS, fosfomycin; GM, gentamicin; TIG, tigecycline; CRBSI, catheter-related bloodstream infection; IAI, intraabdominal infection; RTI, respiratory tract infection; SSTI, skin and soft tissue infection; UTI, urinary tract infection; ICU, intensive care unit; S. marcescens, Serratia marcescens; En. cloacae, Enterobacter cloacae; M. morganii, Morganella morganii.

The included 11 studies enrolled 1,205 patients. The site of bacteremia varied between studies, involving mainly the urinary tract, respiratory tract, and intraabdominal structures or were catheter related. In 6 studies, all patients were infected with Klebsiella pneumoniae, and multiple pathogens were identified in the other 5 studies, although Klebsiella pneumoniae was still the major pathogen (79% to 88%). KPC was the predominant mechanism of carbapenem resistance (70% to 100%) in 6 studies, whereas OXA-48 and metallo-β-lactamases (MBLs) were the main causes of carbapenem resistance in 2 studies and 1 study, respectively. As shown in Table S3, CAZ-AVI was administered in 2% to 52% of patients, most of whom received combination therapy mainly with carbapenem and tigecycline. Antimicrobial agents varied a lot in the control group and included mainly tigecycline- and colistin-containing regimens (from 0% to 81.5% and 3% to 60%, respectively). The most common combination regimen in the control group was colistin + tigecycline.

### Primary and secondary outcomes.

**FIG 2:**
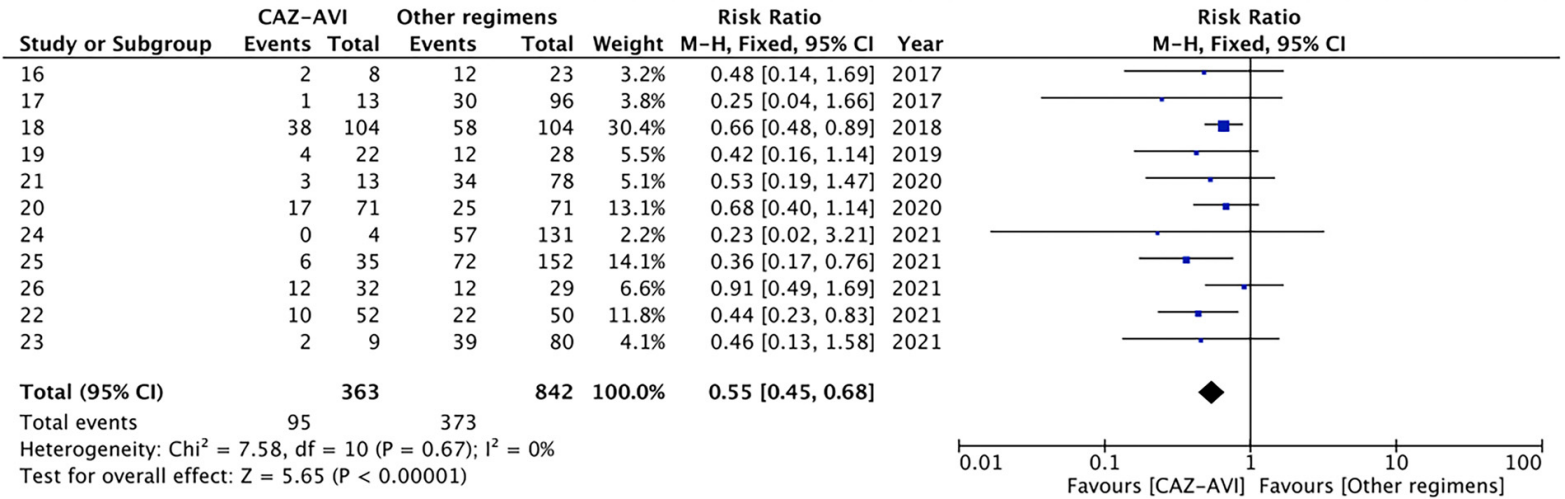
Thirty-day mortality of the CAZ-AVI regimens compared with controls in CRE BSI.

**FIG 3:**
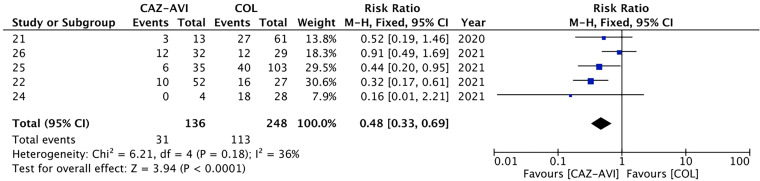
Subgroup analysis of colistin (COL)-containing regimen as a comparator on primary outcome in CRE BSI. Mantel-Haenszel (M-H) are fixed-effect meta-analysis methods using a different weighting scheme that depends on which effect measure is being used.

**(i) Thirty-day mortality.** All-cause 30-day mortality was reported in 11 studies, including 1,205 patients. Compared with the control group, patients in the CAZ-AVI group had a significantly lower 30-day mortality rate (RR = 0.55, 95% CI of 0.45 to 0.68, *I*^2^ = 0%, *P* < 0.00001; Fig. 2). Compared to the treatment of colistin-containing regimens, the CAZ-AVI group showed a lower 30-day mortality rate (RR = 0.48, 95% CI of 0.33 to 0.69, *I*^2^ = 36%, *P* < 0.0001; Fig. 3). In addition, according to the subgroup analysis of carbapenemase, the association of CAZ-AVI treatment with decreased mortality rate was observed both in patients infected with CRE producing KPC (RR = 0.59, 95% CI of 0.46 to 0.75, *I*^2^ = 0%, *P* < 0.0001) and MBL (RR = 0.44, 95% CI of 0.23 to 0.83, *P* = 0.01; Fig. S2).

**(ii) Clinical cure.** The clinical cure rate was reported in 6 studies, including 567 patients. Compared with the control group, patients in the CAZ-AVI group had a significantly higher clinical cure rate (RR = 1.85, 95% CI of 1.57 to 2.18, *I*^2^ = 0%, *P* < 0.00001; Fig. S3).

**(iii) Relapse.** Meta-analysis of 4 studies with 455 patients showed a comparable relapse rate between patients in CAZ-AVI and control groups (RR = 0.69, 95% CI of 0.29 to 1.66, *I*^2^ = 54%, *P* = 0.41; Fig. S4). In sensitivity analyses, exclusion of the study by Tsolaki and colleagues (19) resolved the heterogeneity without changing the result (RR = 1.06, 95% CI of 0.57 to 1.97, *I*^2^ = 0%, *P* = 0.86).

**(iv) Nephrotoxicity.** Pooled results from 5 studies with 380 patients indicated a lower nephrotoxicity rate in the CAZ-AVI group (RR = 0.41, 95% CI of 0.20 to 0.84, *I*^2^ = 2%, *P* = 0.02; Fig. S5).

## DISCUSSION

In this systematic review and meta-analysis of 11 observational studies comparing CAZ-AVI with any other comparators in patients with CRE BSIs, we found that CAZ-AVI was associated with improved all-cause 30-day mortality rate and clinical cure rate and less nephrotoxicity. In addition, the relapse rate was similar between CAZ-AVI and comparators.

CRE has been increasingly reported as a major pathogen in hospital-acquired infections due to, at least in part, worldwide empirical use of carbapenems in severe infections. Despite multiple mechanisms of carbapenem resistance, the production of β-lactamases, especially carbapenemases, is the main resistance mechanism (27). With efficacy against Ambler class A, C, and D β-lactamases, CAZ-AVI is regarded as a drug of choice in CRE infections. Our study demonstrated that, compared with other antibiotics (including colistin), CAZ-AVI treatment was associated with improved clinical cure rate, which might explain the significantly lower mortality rate in patients with CRE BSIs. This was in line with findings of a previous meta-analysis, which also reported statistically higher clinical response rate (RR = 2.11, 95% CI of 1.54 to 2.88) of BSIs in the CAZ-AVI group based on subgroup analysis with a smaller sample size (140 patients in 2 studies) (15).

Moreover, our study suggested the superiority of CAZ-AVI over colistin on the primary outcome in CRE BSIs. Before the availability of novel drugs, including CAZ-AVI, colistin was one of the most frequent drugs used both in monotherapy and combination regimens (7). Furthermore, due to the high case fatality of CRE BSIs, the likelihood of initially appropriate therapy (hazard ratio [HR] of 0.45, 95% CI of 0.33 to 0.62, *P* < 0.0001) has been important to avoid poor outcomes. The IDSA did not identify sufficient evidence to provide recommendations on the treatment of BSIs caused by CRE similar to UTIs (14). As a result, the optimized antimicrobial use among patients with BSIs caused by CRE remains unresolved for antimicrobial stewardship programs. The current result showed improved survival in the CAZ-AVI group compared to the comparators (colistin-based regimen included) in CRE BSIs. Our findings suggest that CAZ-AVI could be used as first-line treatment in patients with known or suspected CRE BSIs.

Due to the lack of available data, we could not perform the subgroup analysis of monotherapy versus combination therapy. However, several recent meta-analyses reported similar mortality rates and microbiological cure rates in patients with infections due to CRE or carbapenem-resistant P. aeruginosa who were treated with CAZ-AVI in monotherapy or combination therapy (28, 29). Moreover, Tumbarello et al. reported a cohort of KPC-producing K. pneumoniae infections treated with CAZ-AVI, and 67.8% of the patients were BSIs (30). No difference was found in 30-day mortality between monotherapy and combination therapy (26.1% versus 25.0%, *P* = 0.79).

Additionally, the high prevalence of KPC-producing CRE in included studies might contribute to the observed survival benefit in our meta-analysis. Unlike KPC, clinical experience in the treatment of OXA-48- and MBL-producing *Enterobacterales* remained limited. As a result, the difference between the efficacy of CAZ-AVI for infections caused by KPC-, OXA-48-, and MBL-producing *Enterobacterales* remains to be elucidated. Carbapenemase subgroup analysis demonstrated the efficacy of CAZ-AVI in KPC-producing *Enterobacterales*. Because of insufficient evidence, no conclusion could be drawn on infection caused by different carbapenemases other than KPC.

The relapse rate was similar in the CAZ-AVI group and comparator group, that is, 10.0% and 15.8%, respectively. Sensitivity analysis suggested that the trial by Tsolaki et al. (19) contributed to the observed heterogeneity. The higher relapse rate (18.2% versus 6 to 10%) might be the result of enrollment of more critically ill patients (i.e., patients with mechanical ventilation) in this study. In addition, the definitions of relapse were different in these studies, which referred to the onset of a second microbiologically documented CRE infection within 30 days, 60 days, or 90 days or index hospitalization after clinical cure of the original CRE infection.

Several limitations of this meta-analysis should be considered. First, included studies were all observational studies with small sample sizes, which were inevitably subject to confounders and bias. Second, the significant diversity of antibiotic regimens in CAZ-AVI and comparator groups in included studies precluded the possibility of recommending one antibiotic regimen over the others, although effort has been made to compare the efficacy between CAZ-AVI and colistin. Third, data on adverse events other than nephrotoxicity were not available.

### Conclusion.

Compared with other antibiotics, CAZ-AVI treatment was associated with lower 30-day mortality, even compared with colistin. Improved clinical cure and nephrotoxicity were also observed in patients with CRE BSIs. In addition, the risk of relapse was comparable. These findings suggested that CAZ-AVI might be considered the drug of choice in selected patients at risk of CRE BSIs. However, these results still await validation by prospective RCTs in the future.

## MATERIALS AND METHODS

### Literature search.

We conducted a literature search of PubMed, EMBASE, and the Cochrane Library from inception until 5 November 2021 to identify potentially relevant studies. The PubMed search strategy was “ceftazidime” and “avibactam” or “AVE1330A” or “Avycaz” or “NXL104” searched both in Medical Subject Headings (MeSH) and relevant keywords (Supplementary materials Search Strategy), which was adapted for EMBASE and Cochrane Library. In addition, complementary searches of potentially relevant articles were manually sought in all of the reference lists of eligible studies. No language restriction was applied.

### Study selection.

Two reviewers (Y.C. and H.-B.H.) independently conducted the literature search and screened the retrieved literature. Studies that compared the efficacy of CAZ-AVI and other antibiotics in patients with CRE BSI and reported primary outcomes (i.e., mortality rate) were eligible, regardless of the study design.

Exclusion criteria were (i) duplicate publications, (ii) no full-text available studies (i.e., conference abstracts), (iii) case reports and case series with no comparators, (iv) studies without extractable data on CRE infections, (v) *in vitro* studies, and (vi) studies not reporting primary outcome. Discrepancies between the reviewers were addressed with a group discussion for a consensus.

### Quality assessment.

The methodological quality of included observational studies was independently assessed by two investigators (Y.C. and H.-B.H.) with the Newcastle-Ottawa scale (NOS) to assess the risk of bias in patient selection, comparability between groups, and exposure or outcome. Studies with NOS scores ≥ 7 were considered high-quality studies. Publication bias was assessed by funnel plot.

### Data extraction.

The following information was extracted from included studies: (i) first author, year of publication, and country where the study was performed; (ii) characteristics of the study (study design, number of subjects); (iii) characteristics of participants (age, sex, type of infection, pathogens, and bacterial resistance); (iv) clinical outcome (mortality rate at 30 days or the end of follow-up, clinical cure and relapse rate) and complication (renal failure).

### Definition and outcomes.

The primary outcome is 30-day mortality (including 28-day mortality), and the secondary outcomes include the clinical cure rate, relapse rate, and nephrotoxicity. In the present report, the clinical cure was defined in terms of resolution of all symptoms and signs of infection. The term relapse refers to the onset of a second infection caused by the original pathogen after the patient had recovered from the initial infection. Nephrotoxicity was defined as a renal failure during treatment.

### Statistical analysis.

Relative risks (RRs) and 95% confidence intervals (CIs) were calculated for dichotomous outcomes of all the relevant studies. Mean differences (MD) and 95% CIs were estimated as the effective results for continuous outcomes. For studies that reported the median as the measure of treatment effect with accompanying interquartile range (IQR), we estimated mean from the median and standard deviations (SD) from IQR or range using the methods described in the previous studies (31). Heterogeneity was tested by using the *I*^2^ statistic. A random effects model was used for results with substantial heterogeneity (*I*^2^ > 50%), whereas a fixed effects model was used in case of insignificant heterogeneity. Whenever significant heterogeneity was present, sensitivity analyses were performed by excluding one trial in each turn to test the influence of a single study on the overall pooled estimate. To test the robustness of our primary outcomes and explore the influence factors, we also conducted subgroup analyses for primary outcomes according to the specified control group of colistin and type of carbapenemases (KPC, metallo-β-lactamases). Review Manager 5.3 (Cochrane Collaboration, Oxford, UK) was used for data analysis.
