# Supplementary material for: Efficacy and Safety of Ceftazidime-Avibactam for the Treatment of Carbapenem-Resistant Enterobacterales Bloodstream Infection: a Systematic Review and Meta-Analysis
Source: Microbiol Spectr. 2022 Apr 4;10(2):e02603-21. doi: 10.1128/spectrum.02603-21 (PMC9045088; doi:10.1128/spectrum.02603-21)
Supplement: SUPPLEMENTAL FILE 1 — Supplemental material. Download SPECTRUM02603-21_Supp_1_seq6.pdf, PDF file, 0.7 MB [file spectrum02603-21_supp_1_seq6.pdf]

Supplementary Table S1 Quality scoring for included cohort studies using New castle-Ottawa Scale (NOS)

First author, year [ref.]	Selection				Comparability		Outcome			Total scores
	Representativeness of the exposed cohort	Selection of the non-exposed cohort	Ascertainment of exposure	Outcome of interest was not present at start of study	On basis of the design	On basis of the analysis	Assessment of outcome	Was follow-up long enough for outcomes to occur?	Adequacy of follow-up of cohorts	
Castón 2017	1	1	1	1	1	0	1	0	0	6
Shields 2018	1	1	1	1	1	0	1	0	0	6
Tumbarello 2019	0	1	1	1	1	0	1	1	1	7
Tsolaki 2019	1	1	1	1	1	0	1	1	0	7
Falcone 2021	1	1	1	1	1	0	1	1	1	8
Hakeam 2021	1	1	1	1	1	0	1	1	0	7

Supplementary Table S2 Quality scoring for included case-control study using New castle-Ottawa Scale (NOS)

First author, year [ref.]	Selection				Comparability		Exposure			Total score
	Case definition	Case representation	Control selection	Control definition	On basis of the design	On basis of the analysis	Assessment of exposure	Same method of ascertainment	Response rate	
Falcone 2020	1	1	1	0	1	0	1	1	1	7
Karaiskos 2020	1	1	1	1	1	1	1	1	0	8
Shen 2021	1	1	1	0	1	1	1	1	0	7
Zhou 2021	1	1	1	1	1	1	1	1	0	8
Chen 2021	1	1	1	0	1	1	1	1	0	7

Supplementary Table S3 Detailed antimicrobial therapy of patients with bloodstream infections caused by CRE

Reference	CAZ-AVI containing-regimen (n, %)	Other regimens (n, %)
Caston 2017	Monotherapy (2/8,25)<br>Combination therapy (6/8,75):<br>AG (7,87.5), CB (3,37.5), FOS (2,25), TIG (2,25), COL (2,25)	Monotherapy (11/23,47.8)<br>Combination therapy (12/23,52.2)
Shields 2017	Monotherapy (8/13,61.5)<br>Combination therapy (5/13,38.4):<br>CAZ-AVI+GM (5,100)	Monotherapy (29/96,30.2):<br>AG (11,37.9), CB (8,27.6), COL (4,13.8), TIG (4,13.8), ciprofloxacin (2,6.9)<br>Combination therapy (67/96,69.8):<br>CB+AG (25,37.3), CB+COL (30,44.8), COL+TIG (3,4.4), AG+TIG (2,3), AG+cefepime (1,1.5), AG+COL+TIG (1,1.5), COL+ATM (1,1.5), COL+cefepime (1,1.5), COL+ciprofloxacin (1,1.5), CB+doxycycline (1,1.5), CB+TIG (1,1.5)
Tumbarello 2019	Monotherapy (22/104,21.2)<br>Combination therapy (82/104,78.8):<br>CAZ-AVI+AK (2,2.4), CAZ-AVI+FOS (7,8.5), CAZ-AVI+TIG (16,19.5), CAZ-AVI+CB (19,23.2), CAZ-AVI+COL (13,15.9), CAZ-AVI+GM (25,30.5)	Monotherapy (27/104, 26):<br>GM (14, 51.9), COL (9, 33.3), Others (4, 14.8)<br>Combination therapy (77/104,74):<br>Double CB (29,37.7), FOS+AK (13,16.9), FOS+GM (11,14.3), GM+CB (11,14.3), COL+FOS (10,13), Others (3,3.9)
Tsolaki 2019	Monotherapy (7/22,31.8)	Monotherapy (1/28,3.6)

	Combination therapy (15/22,68.2):<br>AG (5,33.3), COL (12,80), TIG (8,53.3), FOS (3,20)	Combination therapy (27/28,96.4):<br>AG (9,33.3), COL (24,88.8), TIG (23,85.2), TMP/SMX (2,7.4)
Falcone 2020	CAZ-AVI ± AG/FOS (13/13,100)	Monotherapy (0/78,0)<br>Combination therapy (78/78,100):<br>COL+CB+FOS/AG (61,78.2), TIG+CB (8,10.3), TIG+GM (6,7.7), Double CB (3,3.8)
Karaiskos 2020	CAZ-AVI-containing (71/71,100)	Monotherapy (29/71,40.8):<br>AG (6,20.7), COL (9,31), TIG (14,48.3)<br>Combination therapy (42/71,59.2)
Falcone 2021	Monotherapy (0/52,0)<br>Combination therapy (52/52,100):<br>CAZ-AVI+ATM (52,100)	Monotherapy (4/50,8):<br>COL (2,50), ATM (2,50)<br>Combination therapy (46/50,92):<br>COL+FOS+TIG (7,15.2), COL+FOS (7,15.2), COL+CB (5,10.9), COL+ATM+ BLIBL (4,8.7), COL+GM (1,2.2), COL+ cotrimoxazole (1,2.2), TIG+AG (8,17.4), FOS+AG (5,10.9), TIG+FOS (2,4.3), TIG+CB (1,2.2), ATM+AG (4,8.7), ATM+FOS (1,2.2)
Shen 2021	Monotherapy (4/9, 44.4)	TIG-containing (35/80,43.8), COL-containing (20/80,25), Others (25,31.3)

	Combination therapy (5/9, 55.6):<br>CAZ-AVI+TIG (3,60), CAZ-AVI+COL (1,20), CAZ-AVI+CB (1,20)	
Zhou 2021	Monotherapy (1/4,25)<br>Combination therapy (3/4,75):<br>CAZ-AVI+CB (1,33.3), CAZ-AVI+TIG (2,66.7)	Monotherapy (91/131,69.5):<br>TIG (71,78), CB (10,11), AK (6,6.6), COL (3,3.3), Other (1,1.1)<br>Combination therapy (40/131, 30.5):<br>TIG+COL (21,52.5), TIG+CB (6,15), TIG+AK (6,15), TIG+COL+CB (2,5), TIG+COL+AK (2,5), TIG+CB+AK (2,5), CB+AK (1,2.5)
Hakeam 2021	Monotherapy (9/32,28.1)<br>Combination therapy (23/32,71.9):<br>CB (4,17.4), ATM (7,30.4), Cefepime (1,4.3), AG (3,13), Fluoroquinolones (1,4.3), TIG (8,34.8), Vancomycin (7,30.4)	Monotherapy (0/29,0)<br>Combination therapy (29/29,100):<br>COL(29,100), CB (21,72.4), ATM (1,3.4), Cefepime (2,6.9), BLIBL (2,6.9), AG (6,20.7), Fluoroquinolones (1,3.4), TIG (9,31), Vancomycin (9,31)
Chen 2021	Monotherapy (13/35,37.1)<br>Combination therapy (22/35,62.9):<br>CAZ-AVI+TIG (13,59.1), CAZ-AVI+TIG+COL (9,40.9)	Monotherapy (19/152,12.5):<br>TIG (19,100)<br>Combination therapy (133/152,87.5):<br>TIG+COL (46,34.6), CB+TIG+COL (44,33.1), CB+COL+AG (13,9.8), CB+TIG (14,10.5), CB+AG (16,12)

AG aminoglycosides, AK amikacin, ATM aztreonam, BLIBL Beta-lactamase-inhibiting beta-lactams, CB carbapenems, COL colistin, FOS fosfomycin, GM gentamycin, TIG tigecycline, TMP/SMX Trimethoprim-Sulfamethox

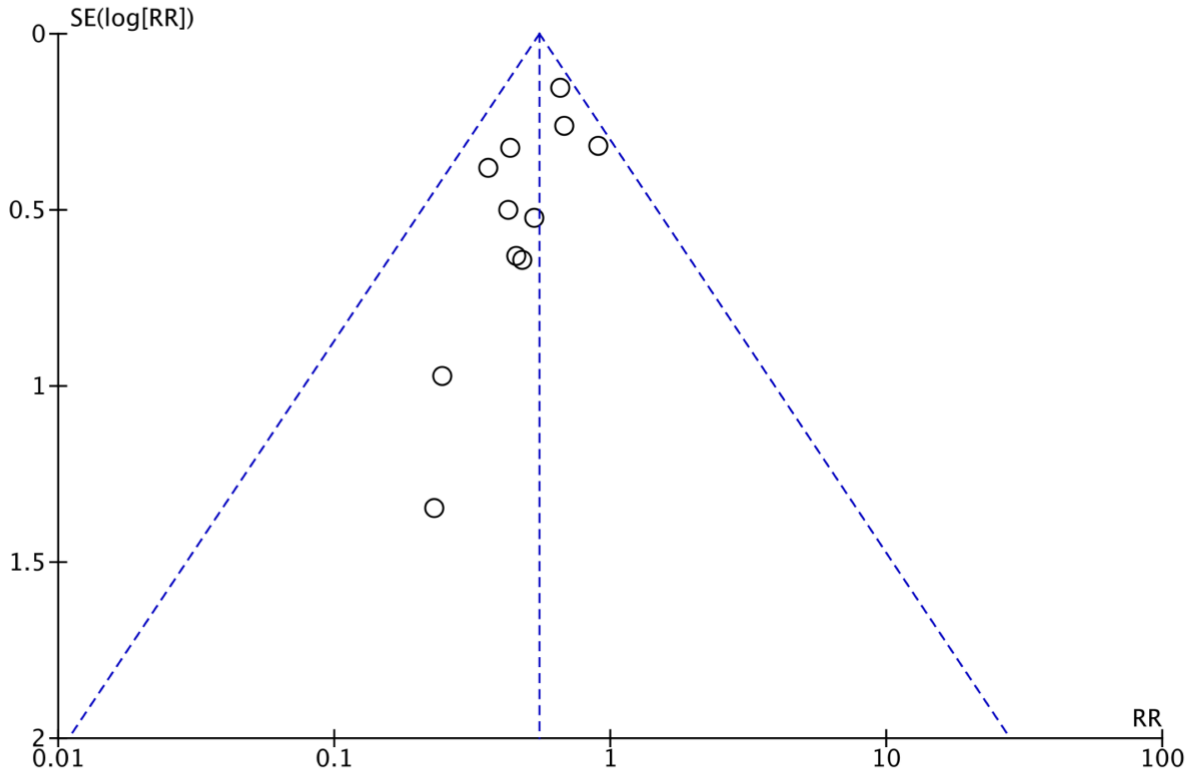

Figure S1. The funnel plot of publication bias

Study or Subgroup	CAZ-AVI Events	CAZ-AVI Total	Other regimens Events	Other regimens Total	Weight	Risk Ratio M-H, Fixed, 95% CI	Year
**1.2.1 KPC**							
Shields 2017	1	13	30	96	5.9%	0.25 [0.04, 1.66]	2017
Tumbarello 2019	38	104	58	104	47.4%	0.66 [0.48, 0.89]	2018
Falcone 2020	3	13	34	78	7.9%	0.53 [0.19, 1.47]	2020
Karaiskos 2020	17	71	25	71	20.4%	0.68 [0.40, 1.14]	2020
**Subtotal (95% CI)**		**201**		**349**	**81.7%**	**0.62 [0.48, 0.80]**	
Total events	59		147				
Heterogeneity: Chi² = 1.24, df = 3 (P = 0.74); I² = 0%							
Test for overall effect: Z = 3.60 (P = 0.0003)							
**1.2.2 MBL**							
Falcone 2021	10	52	22	50	18.3%	0.44 [0.23, 0.83]	2021
**Subtotal (95% CI)**		**52**		**50**	**18.3%**	**0.44 [0.23, 0.83]**	
Total events	10		22				
Heterogeneity: Not applicable							
Test for overall effect: Z = 2.54 (P = 0.01)							
**Total (95% CI)**		**253**		**399**	**100.0%**	**0.59 [0.46, 0.75]**	
Total events	69		169				
Heterogeneity: Chi² = 2.46, df = 4 (P = 0.65); I² = 0%							
Test for overall effect: Z = 4.34 (P < 0.0001)							
Test for subgroup differences: Chi² = 0.99, df = 1 (P = 0.32), I² = 0%							

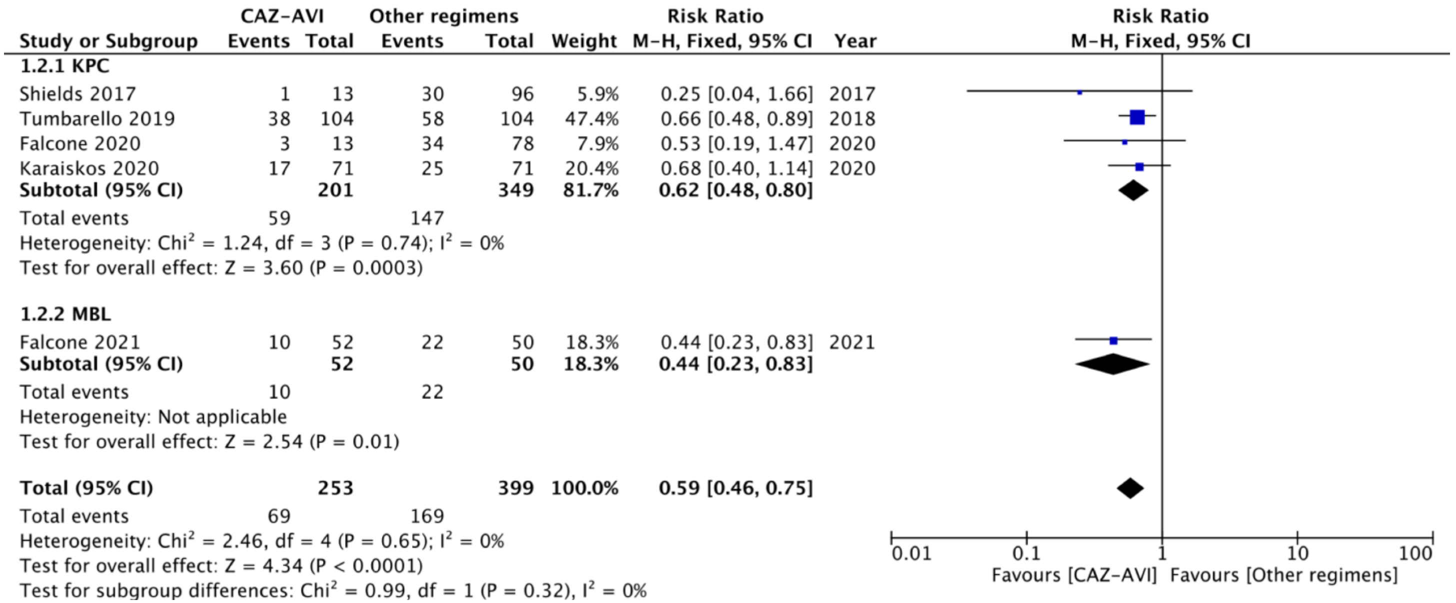

Figure S2. Subgroup analysis of carbapenemase on primary outcome in CRE BSI
MBL Metallo-β-lactamases

	CAZ-AVI		Other regimens			Risk Ratio	
**Study or Subgroup**	**Events**	**Total**	**Events**	**Total**	**Weight**	**M-H, Fixed, 95% CI**	**Year**
Caston 2017	6	8	8	23	5.0%	2.16 [1.08, 4.29]	2017
Shields 2017	11	13	39	96	11.3%	2.08 [1.49, 2.91]	2017
Tsolaki 2019	33	41	19	36	24.6%	1.53 [1.08, 2.15]	2019
Hakeam 2021	15	32	6	29	7.6%	2.27 [1.02, 5.05]	2021
Chen 2021	25	35	48	152	21.8%	2.26 [1.65, 3.10]	2021
Falcone 2021	39	52	24	50	29.7%	1.56 [1.13, 2.17]	2021
**Total (95% CI)**		**181**		**386**	**100.0%**	**1.85 [1.57, 2.18]**	
Total events	129		144				

Heterogeneity: $Chi^2 = 4.72$, df = 5 (P = 0.45); $I^2 = 0\%$
Test for overall effect: Z = 7.29 (P < 0.00001)

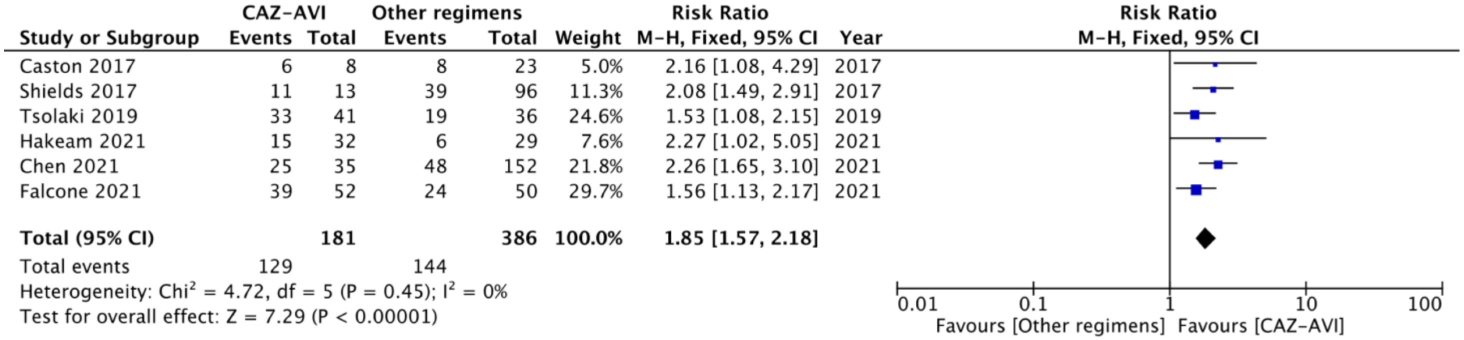

Figure S3. Clinical cure rate of CAZ-AVI regimens compared with controls in CRE BSI

	CAZ-AVI		Other regimens			Risk Ratio	
**Study or Subgroup**	**Events**	**Total**	**Events**	**Total**	**Weight**	**M-H, Random, 95% CI**	**Year**
Shields 2017	2	13	17	96	22.2%	0.87 [0.23, 3.34]	2017
Tumbarello 2019	10	104	9	104	32.3%	1.11 [0.47, 2.62]	2018
Tsolaki 2019	2	41	12	36	20.9%	0.15 [0.04, 0.61]	2019
Hakeam 2021	5	32	4	29	24.6%	1.13 [0.34, 3.82]	2021
**Total (95% CI)**		**190**		**265**	**100.0%**	**0.69 [0.29, 1.66]**	
Total events	19		42				

Heterogeneity: $Tau^2 = 0.43$; $Chi^2 = 6.55$, df = 3 (P = 0.09); $I^2 = 54\%$
Test for overall effect: Z = 0.82 (P = 0.41)

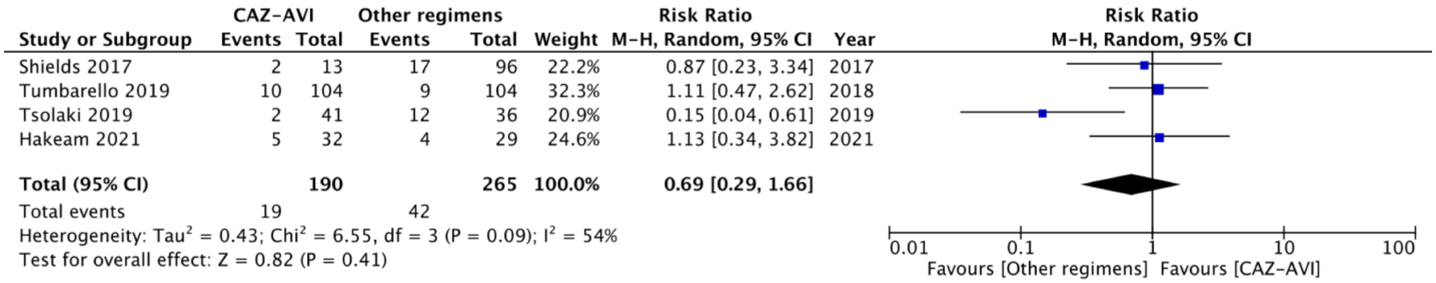

Figure S4. Relapse rate of CAZ-AVI regimens compared with controls in CRE BSI

	CAZ-AVI		Other regimens			Risk Ratio	
**Study or Subgroup**	**Events**	**Total**	**Events**	**Total**	**Weight**	**M-H, Fixed, 95% CI**	**Year**
Shields 2017	0	13	8	96	9.2%	0.41 [0.02, 6.68]	2017
Caston 2017	2	8	7	23	15.5%	0.82 [0.21, 3.17]	2017
Tsolaki 2019	2	41	4	36	18.2%	0.44 [0.09, 2.26]	2019
Falcone 2021	1	52	10	50	43.6%	0.10 [0.01, 0.72]	2021
Hakeam 2021	3	32	3	29	13.5%	0.91 [0.20, 4.14]	2021
**Total (95% CI)**		**146**		**234**	**100.0%**	**0.41 [0.20, 0.84]**	
Total events	8		32				

Heterogeneity: $Chi^2 = 4.06$, df = 4 (P = 0.40); $I^2 = 2\%$
Test for overall effect: Z = 2.42 (P = 0.02)

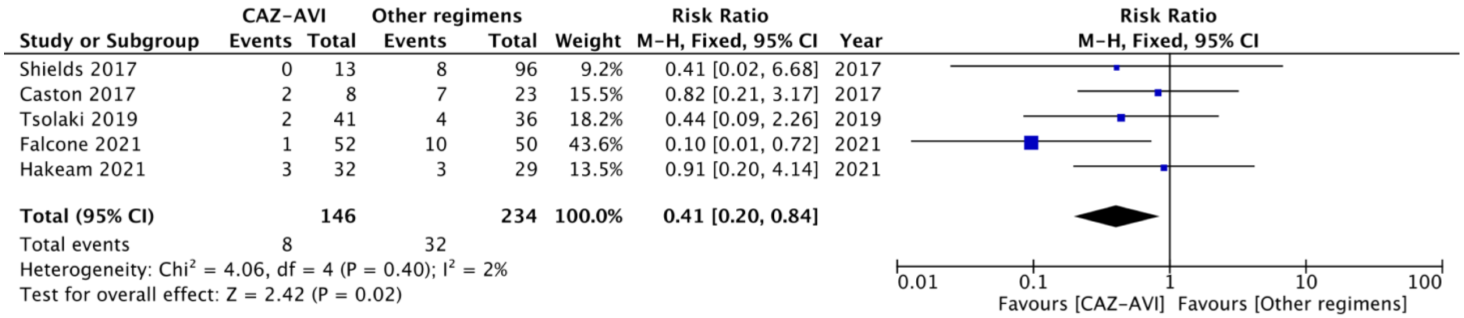

Figure S5. Nephrotoxicity of the CAZ-AVI regimens compared with control in CRE BSI

Search Strategy

Pubmed

Search (((("avibactam, ceftazidime drug combination" [Supplementary Concept]) OR ((((((ceftazidime/avibactam[Title/Abstract]) OR ceftazidime-avibactam[Title/Abstract]) OR avibactam-ceftazidime[Title/Abstract]) OR Avycaz[Title/Abstract]) OR NXL104[Title/Abstract]) OR AVE1330A[Title/Abstract]))) AND (((((((((((((((carbapenem resist*[Title/Abstract]) OR carbapenemase producing[Title/Abstract]) OR carbapenem resistance[Title/Abstract]) OR KPC[Title/Abstract]) OR Metallo Lactamase[Title/Abstract]) OR Metallo beta lactamase[Title/Abstract]) OR VIM-producing[Title/Abstract]) OR VIM produc*[Title/Abstract]) OR IMP-producing[Title/Abstract]) OR IMP produc*[Title/Abstract]) OR NDM-producing[Title/Abstract]) OR NDM produc*[Title/Abstract]) OR OXA[Title/Abstract]) OR CRE[Title/Abstract]) OR Carbapenem-resistant[Title/Abstract])) AND ((risk[Title/Abstract] OR risk[MeSH:noexp] OR mortality[Title/Abstract] OR mortality[MeSH:noexp] OR cohort[Title/Abstract]))

Embase

#12.	#7 AND #10 AND #11	166	5 Nov 2021
#11.	'risk':ab,ti OR 'mortalit':ab,ti OR 'cohort':ab,ti	4,070,182	5 Nov 2021
#10.	#8 OR #9	62,797	5 Nov 2021
#9.	'carbapenem resist*':ab,ti OR 'carbapenemase producing':ab,ti OR 'carbapenem resistance':ab,ti OR 'kpc':ab,ti OR 'metallo lactamase':ab,ti OR 'metallo beta lactamase':ab,ti OR 'vim-producing':ab,ti OR 'vim produc*':ab,ti OR 'imp-producing':ab,ti OR 'imp produc*':ab,ti OR 'ndm-producing':ab,ti OR 'ndm produc*':ab,ti OR 'oxa':ab,ti OR 'cre':ab,ti OR 'carbapenem-resistant':ab,ti	62,227	5 Nov 2021
#8.	'carbapenem-resistant enterobacteriaceae'/exp	3,442	5 Nov 2021
#7.	#1 OR #2 OR #3 OR #4 OR #5 OR #6	1,801	5 Nov 2021

#6.	'ave1330a':ab,ti	6	5 Nov 2021
#5.	'nxl104':ab,ti	55	5 Nov 2021
#4.	'avycaz':ab,ti	10	5 Nov 2021
#3.	'ceftazidime avibactam':ab,ti	1,136	5 Nov 2021
#2.	'avibactam plus ceftazidime'/exp	1,593	5 Nov 2021
#1.	'avibactam ceftazidime':ab,ti	23	5 Nov 2021

Cochrane

#1 (avibactam ceftazidime):ti,ab,kw OR (Avycaz):ti,ab,kw OR (NXL104):ti,ab,kw OR (AVE1330A):ti,ab,kw OR (avibactam, ceftazidime drug combination):ti,ab,kw (Word variations have been searched) 91

#2 MeSH descriptor: [Carbapenem-Resistant Enterobacteriaceae] explode all trees 4

#3 (carbapenem resist*):ti,ab,kw OR (carbapenemase producing):ti,ab,kw OR (carbapenem resistance):ti,ab,kw OR (KPC):ti,ab,kw OR (Metallo Lactamase):ti,ab,kw (Word variations have been searched) 368

#4 (Metallo beta lactamase):ti,ab,kw OR (VIM-producing):ti,ab,kw OR (VIM produc*):ti,ab,kw OR (IMP-producing):ti,ab,kw OR (IMP produc*):ti,ab,kw (Word variations have been searched) 774

#5 (NDM-producing):ti,ab,kw OR (NDM produc*):ti,ab,kw OR (OXA):ti,ab,kw OR (CRE):ti,ab,kw OR (Carbapenem-resistant):ti,ab,kw (Word variations have been searched)1127

#6 #2 OR #3 OR #4 OR #5 2047

#7 #1 AND #6 24
